# Expressions of Matrix Metalloproteinases 2, 7, and 9 in Carcinogenesis of Pancreatic Ductal Adenocarcinoma

**DOI:** 10.1155/2016/9895721

**Published:** 2016-06-26

**Authors:** Katarzyna Jakubowska, Anna Pryczynicz, Joanna Januszewska, Iwona Sidorkiewicz, Andrzej Kemona, Andrzej Niewiński, Łukasz Lewczuk, Bogusław Kędra, Katarzyna Guzińska-Ustymowicz

**Affiliations:** ^1^Department of Pathomorphology, Comprehensive Cancer Center, 15-027 Białystok, Poland; ^2^Department of General Pathomorphology, Medical University of Białystok, 15-269 Białystok, Poland; ^3^Department of Reproduction and Gynecological Endocrinology, Medical University of Białystok, 15-276 Białystok, Poland; ^4^Department of Rehabilitation, Medical University of Białystok, 15-276 Białystok, Poland; ^5^2nd Department of General and Gastroenterological Surgery, Medical University of Białystok, 15-276 Białystok, Poland

## Abstract

Pancreatic ductal adenocarcinoma (PDAC) is a highly fatal disease, usually diagnosed in an advanced stage which gives a slight chance of recovery. Matrix metalloproteinases (MMPs) are a family of zinc-dependent endopeptidases that participate in tissue remodeling and stimulate neovascularization and inflammatory response. The aim of the study was to evaluate the expression of MMP-2, MMP-7, and MMP-9 in normal ducts, tumor pancreatic adenocarcinoma cells, and peritumoral stroma in correlation with clinicohistopathological parameters. The study material was obtained from 29 patients with pancreatic ductal adenocarcinoma. The expressions of MMP-2, MMP-7, and MMP-9 were performed by immunohistochemical technique. Microvessel density (MVD) was visualized by special immunostaining. The expressions of MMP-2, MMP-7, and MMP-9 were mainly observed in tumor cells and peritumoral stroma. MMP-2 expression in cancer cells was correlated with female gender, stronger inflammation, and histopathological type of cancer (*R* = 0.460, *p* = 0.013; *R* = 0.690, *p* = 0.0001; *R* = −0.440, *p* = 0.005, resp.). The expression of MMP-7 in tumor cells was found to positively correlate with the presence of necrosis and negatively correlate with MVD (*R* = 0.402, *p* = 0.031; *R* = −0.682, *p* = 0.000). We also showed that positive MMP-9 expression in tumor cells was associated with MVD (*R* = 0.368, *p* = 0.084); however, it was not statistically significant. Our results demonstrate that MMP-2, MMP-7, and MMP-9 expressions correlate with various morphological features of the PDAC tumor such as inflammation, necrosis, and formation of the new blood vessels.

## 1. Introduction

Pancreatic ductal adenocarcinoma (PDAC) is a highly fatal disease, usually diagnosed in an advanced stage which gives a slight chance of recovery. Pancreatic cancer is characterized by a rapid course, poor prognosis, and high mortality since most patients are diagnosed with metastases to lymph nodes and distant organs [1]. This increases with age and is closely associated with genetic factors [2].

Matrix metalloproteinases (MMPs) are a family of zinc-dependent endopeptidases [3]. MMPs are produced by connective tissue cells (fibroblasts), leukocytes, macrophages, and endothelial cells [4]. They are involved in degradation of the extracellular matrix and have been implicated in physiological processes. MMPs are involved in supporting tissue remodeling and are required for the skeletal development. They stimulate neovascularization, both in physiological and in pathological conditions, for example, in tumors [3]. MMPs can regulate vascular stability and permeability in response to tissue injury [5]. Metalloproteinases are responsible for proper migration of cells involved in the inflammatory response [6]. They allow the cells to move into the damaged tissue and to release cytokines and their receptors through their ability to destroy the basement membrane. It has been shown that metalloproteinases destroy interleukin-2 receptors on T cells which mute the immune response against tumor cells. The imbalance between the activity of MMPs and their tissue inhibitors (TIMPs) has been attributed to the ability of cancer cells to migrate [7]. Recent studies have confirmed that the overexpression of MMPs in tumor and stromal cells in various cancers was associated with tumor invasion and progression [8]. Moreover, MMPs released from distant organs, along with tumor cell growth factors, can participate in premetastatic niche formation and metastasis [9, 10].

Therefore, the aim of our study was to evaluate the immunohistochemical expressions of MMP-2, MMP-7, and MMP-9 in the normal pancreas, pancreatic adenocarcinoma tumor cells, and stromal cells in correlation with clinicopathological features.

## 2. Material and Methods

### 2.1. Materials

The study material was obtained from 29 patients (23 men, 6 women; 14 patients aged ≤60 and 15 aged >60) with pancreatic ductal adenocarcinoma (PDAC), operated on in the 2nd Department of General Surgery and Gastroenterology, Medical University of Białystok. Patients received neither preoperative cancer therapy nor inflammation therapy.

The study was performed in conformity with the Declaration of Helsinki for Human Experimentation and received approval by the Local Bioethics Committee of the Medical University of Białystok (number R-I-002/167/2013).

### 2.2. Histopathological Examination

The routine histopathological assessment of the sections (stained with H+E) was conducted with reference to the histological type, malignancy grade (G), clinicopathological pT status, regional lymph node involvement, and the presence of distant metastases. Inflammation was defined as mild when it consisted of <10 immune cells per 10 hpf under 100x magnification; as moderate when it consisted of 10 to 50 immune cells; and as severe when it consisted of > 50 immune cells [11, 12]. Desmoplasia was classified as poor when it occupied <25% of tumor area observed in 10 hpf under 100x magnification, whereas it was predominant for >25%. Hemorrhage was measured under 400x magnification and defined as single (one focus) or numerous (more than one hemorrhagic focus). Necrosis in the central tumor was graded as absent (none), weak/focal (<10% of tumor area), moderate (10–30%), or strong/extensive (>30%) [13].

### 2.3. Assessment of Vessel Formation

Microvessel density (MVD) was visualized by special immunostaining of collagen type IV. Since protein can be expressed on the mesenchymal components and epithelial basal laminae, to prevent misdiagnosis, we analyzed only vascular structures with distinct (slot-like or tubular) lumens showing positively stained endotheliocyte layers. They were counted as a number of intratumoral microvessels per unit area of the tumor, subjectively selected from the most vascularized areas (5 hpf under 200x magnification) [14]. The PDAC tumors were divided into two groups, with low or high MVD. The cutoff value was the mean MVD. The mean MVD of the tumor tissue was 5.28 vessels (range: 0–21), confirming the histopathological features of hypovascular pancreatic tumors.

### 2.4. Immunohistochemical Analysis

Formalin-fixed and paraffin-embedded tissue specimens were cut on a microtome into 4 *μ*m sections. The sections were deparaffinized in xylenes (CHEM^*∗*^115208603; Chempur, Poland) and hydrated in alcohols. To visualize the antigens of MMP-2, MMP-7, and MMP-9, the sections were heated in a microwave oven for 20 min in EDTA buffer (pH = 9.0) (EDTA buffer, Antigen Retriever; E1161; Sigma-Aldrich Co., MO, USA). Then, they were incubated with 3% hydrogen peroxide solution for 20 min in order to block endogenous peroxidase. Next, incubation was performed with anti-human antibodies against monoclonal antibody of matrix metalloproteinase 2 (clone 17B11; Novocastra, UK; dilution 1 : 60), mouse monoclonal antibody of matrix metalloproteinase 7 (clone 111433; R&D Systems, USA; dilution 1 : 75), and mouse monoclonal antibody of matrix metalloproteinase 9 (clone 15W2; Novocastra, UK, dilution 1 : 80) during a period of 1 hour at room temperature. The reaction was carried out using the streptavidin-biotin system (Biotinylated Secondary Antibody, Streptavidin-HRP, Novocastra, UK). A color reaction for peroxidase was developed with chromogen DAB (DAB, Novocastra, UK). The negative control section was incubated instead of the primary antibody. All section slides were counterstained with hematoxylin.

Immunohistochemical staining was evaluated by two independent pathologists who were blinded to the clinical information. A positive reaction was observed in the cytoplasm of the normal pancreas, pancreatic ductal adenocarcinoma, and peritumoral stroma. Due to the small study group, immune cells and fibroblasts were analyzed jointly. The expression of proteins was evaluated and defined as positive (reaction present in >25% of normal ductal cells, tumor cells, or peritumoral stroma components) or negative (lack of reaction or reaction present in <25% of normal ductal cells, tumor cells, or peritumoral stroma components). The percentage of the reaction-positive cells was calculated in 500 neoplastic cells in each study sample at 400x magnification.

### 2.5. Statistical Analysis

Statistical analysis was conducted using Statistica 10.0 (StatSoft, Kraków, Poland). In order to compare the two groups, the parameters were calculated by the Chi-quadrate test. Correlations between the parameters were calculated by Spearman's correlation coefficient test. A *p* value of <0.05 was considered statistically significant.

## 3. Results

### 3.1. Histopathological Findings

Pancreatic adenocarcinomas were moderately differentiated (G2) in 25/29 cases and poorly differentiated (G3) in 4/29 cases. They were classified as mucinous in 3/29 cases and as nonmucinous in 26/29 cases. We noted lymph node involvement in 12/29 cases and metastases to distant organs (liver, intestine) in 9/29 cases. In addition, we assessed the degree of inflammation, desmoplasia, necrosis, and foci of hemorrhage. We observed a weak inflammatory response in 9 cases, moderate response in 10 cases, and strong response in 10 cases. Tumors with poor and prominent fibrosis were noted in 12 and 17 cases, respectively. Hemorrhage was numerous in 5 cases and single in 10 cases. Pancreatic adenocarcinomas were associated with weak or moderate necrosis in 13 cases.

### 3.2. Expression of Matrix Metalloproteinases (MMP-2, MMP-7, and MMP-9) in Normal Ducts, Pancreatic Adenocarcinoma Tumor Cells, and Stromal Cells

**Figure 1:**
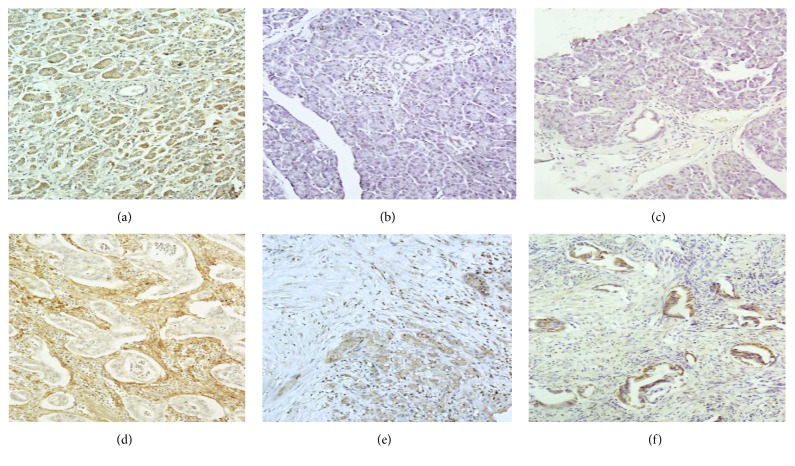
Immunohistochemical expressions of MMP-2, MMP-7, and MMP-9 in normal ducts, tumor cells, and peritumoral stroma of PDAC. The lack of MMP-2 (a), MMP-7 (b), and MMP-9 (c) expressions was found in normal ducts. Positive reaction of MMP-9 (d) was noted in the cytoplasm of pancreatic adenocarcinoma cells. MMP-2 (d) overexpression was observed in the peritumoral stroma in the majority of PDAC cases and color reaction of MMP-7 was observed in the cytoplasm of both cancer cells and the stroma (e). Positive reaction of MMP-9 was noted in the cytoplasm of pancreatic adenocarcinoma cells (f).

The expression of MMP-2 was predominantly present in tumor cells and stroma (55.17% and 79.31%, resp.) in comparison to its absence in the normal pancreas (96.55%). In contrast, the positive expression of MMP-7 was observed mainly in tumor cells (96.55%), less in the stromal cells (55.17%), as compared to the normal pancreas (93.10%). Immunohistochemical assessment of MMP-9 in patients with PDAC showed the protein presence in tumor cells in 44.83% of cases and its absence in normal pancreatic ducts and stroma (100% and 75.87%, resp.) (Figure 1). Furthermore, statistical analysis showed a significant difference between the expressions of MMP-2 and MMP-7 in normal and tumor tissues (*p* = 0.0001). In addition, the expression of MMP-7 in tumor cells differed statistically significantly from that noted in the peritumoral stroma (*p* = 0.005). The differences in MMP expressions (MMP-2, MMP-7, and MMP-9) in normal tissue, tumor cells, and stroma are shown in Table 1.

### 3.3. The Correlation between the Expression of Matrix Metalloproteinases (MMP-2, MMP-7, and MMP-9) in Tumors of PDAC and Clinicopathological Features

The analysis of the expression of matrix metalloproteinases in a variety of tissues and selected anatomical clinical factors demonstrated a correlation between MMP-2 expression in tumor and female gender (*R* = 0.460, *p* = 0.013). A strong positive correlation was noted between MMP-2 in tumor tissue and the presence of stronger inflammation (*R* = 0.690, *p* = 0.0001). MMP-2 expression in stromal cells was found to negatively correlate with the nonmucinous type of PDAC (*R* = −0.440, *p* = 0.005). Positive expression of MMP-2 was more frequent in patients over 60 years of age, although it was not statistically significant. MMP-7 expression in tumor cells revealed a positive association with more frequent occurrence of necrosis in the main mass of tumor (*R* = 0.402, *p* = 0.031) and a negative correlation with the lower index of MVD (*R* = −0.681, *p* = 0.000). Positive expression of MMP-7 was accompanied by frequent changes indicating the presence of necrosis. The expressions of matrix metalloproteinases were not correlated with malignancy grade, fibrosis degree, the incidence of hemorrhage, or the presence of metastases to lymph nodes and distant organs (Tables 2 and 3).

## 4. Discussion

PDAC has poor prognosis and patients' survival time is estimated to be several months. The research into mechanisms of the outstanding malignancy and invasiveness of tumor cells is still being conducted [15]. It has been proven that metalloproteinases are involved in different phases of tumor development, such as extracellular matrix degradation, neovascularization, inhibition of inflammatory cell migration, and metastasis formation in the lymph nodes and distant organs [3, 5, 9, 15]. Their role in the above mechanisms has been investigated in various types of the digestive system tumors located in the colorectum, the stomach, and the liver [16–19].

In our study, we noted a positive expression of MMP-2 in 55.17% of tumor cells and in 79.31% of the stroma, which was significantly higher than in the normal pancreas (3.45%). Giannopoulos et al. [17] also showed the presence of MMP-2 in cancer cells in most cases of PDAC. In turn, Gress et al. [18] demonstrated that the overexpression of MMP-2 was significantly higher in tumor cells than in the stroma. We also reported a positive correlation between the expression of MMP-2 in tumor cells and inflammation. This may suggest that MMP-2 protein is secreted from tumor cells to the stroma where it modifies the immune response cells. In our research, MMP-2 expression was significantly more frequent in the nonmucinous type of PDAC. Histologically, the nonmucinous type of pancreatic cancer is characterized by tubular structures generally located in the rich desmoplastic stroma. Tumor cells of the mucinous type have the ability to produce a large amount of mucus that fills in the tumor microenvironment and may limit the development of connective tissue. The results of our small study suggest that MMP-2 expression correlates with the histopathological type of PDAC and that its expression may be involved in the regulation of the inflammatory response.

MMPs are involved in the degradation of ECM leading to promotion of cancer cell invasion. The smallest family of MMPs, namely, MMP-7, shows the greatest proteolytic activity [19]. In our study, the positive MMP-7 expression was present in most cases in tumor cells, in more than half of the cases in the stroma, and in only two cases in normal pancreatic ducts. Crawford et al. [20] and Li et al. [21] showed the presence of MMP-7 expression in the cytoplasm of most tumor cells and its absence in normal pancreatic ducts. Our statistical analysis demonstrated a significant correlation between MMP-7 expression in PDAC cells and a positive reaction in stromal cells as well as in normal ducts. These results are consistent with the observations of Jones et al. [22]. In contrast, Yamamoto et al. [23] reported an increase in MMP-7 expression in tumor nests located in the front of PDAC tumors. Tan et al. [24] showed that MMP-7 overexpression in the tumor front was present much less frequently than in the center of the tumor mass. In our study, the positive expression of MMP-7 in PDAC tumors was associated with the presence of necrotic lesions. Other studies have confirmed that MMP-7 shows proteolytic activity, participates in cell dissociation through disruption of tight junction structures, determines tumor dissociation from the primary tumor, and stimulates cancer cell invasion [25, 26]. Moreover, we observed a strong, negative relationship between MMP-7 expression in tumor cells and MVD. MMP-7 can cleave collagen IV which constitutes the skeleton of the basement membranes on blood vessels and the ECM components. In turn, MMP-7 may lead to the degradation of the tumor stroma, decay of current vessels, and the inhibition of neovascularization. As a result of these processes, necrotic lesions were observed in tumor mass and hypovascular features of PDAC tumors were noted. Our findings suggest that MMP-7 expression in PDAC cancer cells may contribute to the degradation of the peritumoral stroma, thus facilitating tumor cell invasion and metastasis.

MMP-9 is considered to be a strong factor stimulating the secretion of proangiogenic factors, such as vascular endothelial growth factor (VEGF) which participates in the new blood vessel formation [26, 27]. The study of mouse model has confirmed that tumor cells in MMP-9+/+ mice produce bigger pancreatic tumors with high index of MVD [24]. Moreover, Huang et al. [28] also demonstrated an increased incidence of cancer and tumor size in MMP-9+/+ mice, which was associated with a high index of MVD and increased macrophage infiltration. We noted positive expression of MMP-9 in the cytoplasm of tumor cells and in the stroma of patients with PDAC. Our observations are consistent with those reported by Giannopoulos et al. [17] and Gress et al. [18]. Statistical analysis confirmed that the positive expression of MMP-9 only in the tumor cells showed a growing tendency in MVD. These observations suggest that MMP-9 has an angiogenic property in comparison with much stronger desmoplastic activity of MMP-2 and proteolytic activity of MMP-7 in the tumor stroma in PDAC tumors.

In conclusion, our findings confirmed that MMP-2, MMP-7, and MMP-9 may take part in various morphological features of PDAC tumor. MMP-2 is mainly responsible for the regulation of inflammatory response. MMP-7 takes part in the degradation of the peritumoral stroma whereas MMP-9 may have an impact on the formation of the new blood vessels. In our opinion, immunohistochemical evaluation of the study metalloproteinases in the tissue of patients with PDAC may help to better understand the morphology and development of PDAC tumors. Our observations need to be confirmed in a larger group of PDAC tumors.

## Figures and Tables

**Table 1:** Expressions of MMP-2, MMP-7, and MMP-9 in normal pancreas, cancer cells, and stroma.

	MMP-2		MMP-7		MMP-9	
	Negative	Positive	Negative	Positive	Negative	Positive
Normal tissue	28 (96.55%)	1 (3.45%)	27 (93.10%)	2 (6.90%)	29 (100%)	0 (0%)
Cancer cells	13 (44.83%)	16 (55.17%)^*∗*^	1 (3.45%)	28 (96.55%)^*∗∗*^	16 (55.17%)	13 (44.83%)
Tumor stroma	6 (20.69%)	23 (79.31%)	13 (44.83%)	16 (55.17%)^*∗∗∗*^	22 (75.87%)	7 (24.13%)

^*∗*^MMP-2 in cancer cells versus MMP-2 in normal tissue, *p* = 0.0001.

^*∗∗*^MMP-7 in cancer cells versus MMP-7 in normal tissue, *p* = 0.0001.

^*∗∗∗*^MMP-7 in cancer cells versus MMP-7 in tumor stroma, *p* = 0.005.

**Table 2:** Correlations between MMP-2, MMP-7, and MMP-9 expressions in tumor cells and clinicopathological parameters.

Variables	Patients, *N* (%)	MMP-2		MMP-7		MMP-9	
		*R*	*p* value	*R*	*p* value	*R*	*p* value
*Gender*							
Male	23 (79.3)	0.460	**0.013**	−0.047	0.814	−0.042	0.804
Female	6 (20.7)						
*Age*							
≤60	14 (48.3)	−0.012	0.949	0.339	0.071	−0.383	**0.046**
>60	15 (51.7)						
*Inflammation*							
Absent	2 (6.9)	0.690	<**0.001**	0.053	0.782	0.081	0.666
Weak	9 (31.0)						
Moderate	10 (34.5)						
Strong	8 (27.6)						
*Desmoplasia*							
Poor	12 (41.4)	0.016	0.931	0.215	0.262	0.135	0.491
Prominent	17 (58.6)						
*Foci of hemorrhage*							
Absent	14 (48.3)	0.088	0.648	0.161	0.403	−0.271	0.162
Single	10 (34.5)						
Numerous	5 (17.2)						
*Necrosis*							
Absent	16 (55.2)	−0.007	0.968	0.402	**0.031**	0.084	0.668
Weak	7 (24.1)						
Moderate	6 (20.7)						
Strong	0 (0.0)						
*MVD*							
Low	15 (51.7)	0.072	0.738	**−0.682**	**<0.001**	0.368	0.084
High	14 (48.3)						
*Adenocarcinoma type*							
Nonmucinous	26 (89.7)	−0.193	0.314	−0.139	0.495	−0.081	0.681
Mucinous	3 (10.3)						
*Grade of malignancy*							
G2	25 (86.2)	−0.156	0.417	−0.036	0.850	−0.129	0.512
G3	4 (13.8)						
*Lymph node involvement*							
Absent	17 (58.6)	0.024	0.899	−0.261	0.172	0.033	0.866
Present	12 (41.4)						
*Distant metastases*							
Absent	20 (69.0)	0.014	0.940	−0.193	0.313	−0.081	0.681
Present	9 (31.0)						

**Table 3:** Correlations between MMP-2, MMP-7, and MMP-9 expressions in peritumoral stroma cells and clinicopathological parameters.

Variables	Patients, *N* (%)	MMP-2		MMP-7		MMP-9	
		*R*	*p* value	*R*	*p* value	*R*	*p* value
*Gender*							
Male	23 (79.3)	−0.051	0.793	−0.603	0.060	−0.237	0.215
Female	6 (20.7)						
*Age*							
≤60	14 (48.3)	0.199	0.299	0.029	0.879	−0.261	0.170
>60	15 (51.7)						
*Inflammation*							
Absent	2 (6.9)	0.263	0.167	0.132	0.494	−0.042	0.826
Weak	9 (31.0)						
Moderate	10 (34.5)						
Strong	8 (27.6)						
*Desmoplasia*							
Poor	12 (41.4)	0.033	0.864	−0.129	0.505	0.189	0.325
Prominent	17 (58.6)						
*Foci of hemorrhage*							
Absent	14 (48.3)	−0.198	0.302	−0.010	0.958	−0.164	0.395
Single	10 (34.5)						
Numerous	5 (17.2)						
*Necrosis*							
Absent	16 (55.2)	−0.196	0.306	−0.050	0.796	0.121	0.529
Weak	7 (24.1)						
Moderate	6 (20.7)						
Strong	0 (0.0)						
*MVD*							
Low	15 (51.7)	−0.220	0.300	−0.022	0.916	−0.046	0.829
High	14 (48.3)						
*Adenocarcinoma type*							
Nonmucinous	26 (89.7)	−0.440	**0.005**	0.106	0.584	−0.194	0.313
Mucinous	3 (10.3)						
*Grade of malignancy*							
G2	25 (86.2)	−0.130	0.500	0.125	0.518	−0.021	0.912
G3	4 (13.8)						
*Lymph node involvement*							
Absent	17 (58.6)	−0.021	0.910	−0.176	0.360	−0.105	0.586
Present	12 (41.4)						
*Distant metastases*							
Absent	20 (69.0)	0.101	0.602	−0.106	0.582	−0.224	0.241
Present	9 (31.0)						
